# Supplementary figures and images for: Using deep learning to decipher the impact of telomerase promoter mutations on the dynamic metastatic morpholome
Source: PLoS Comput Biol. 2024 Jul 30;20(7):e1012271. doi: 10.1371/journal.pcbi.1012271 (PMC11288469; doi:10.1371/journal.pcbi.1012271)

A

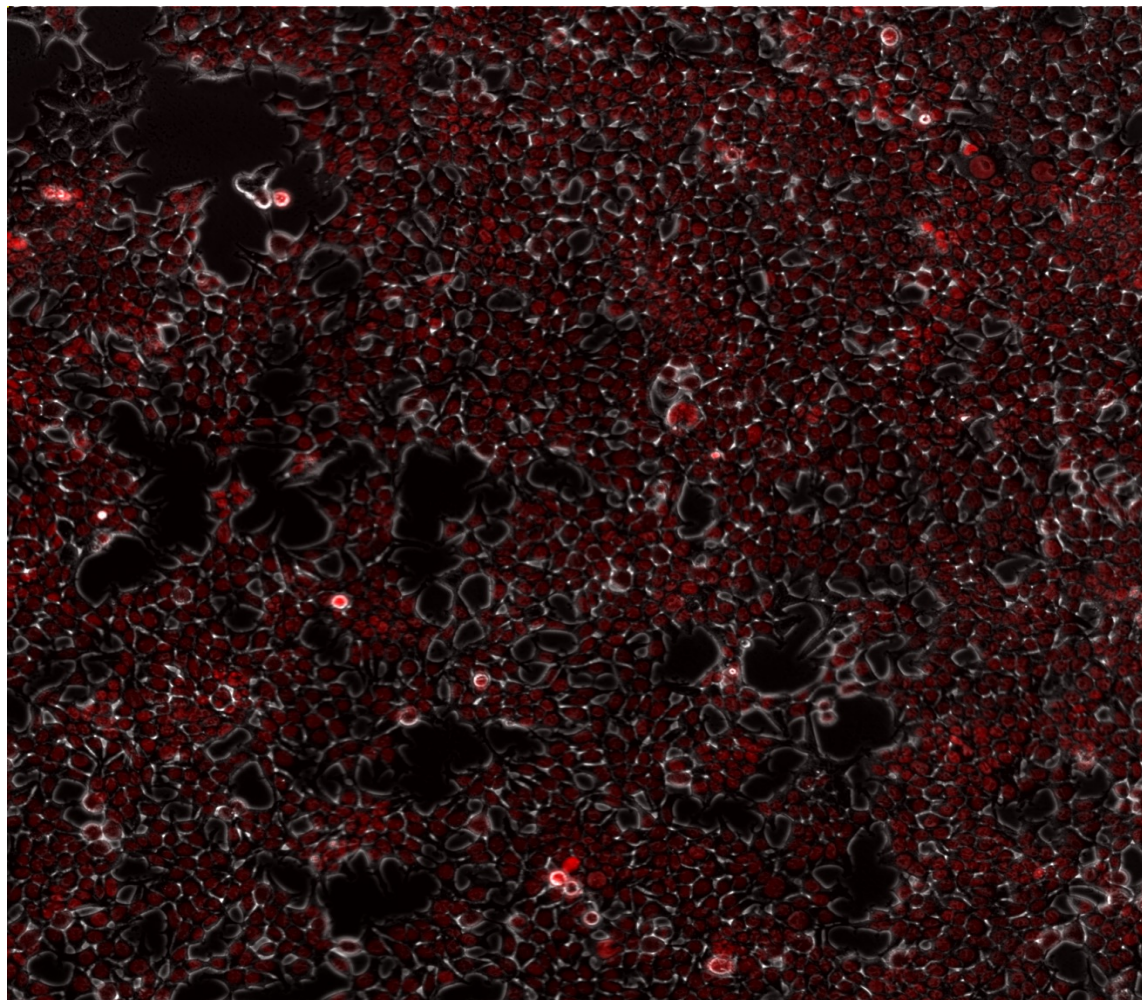

B

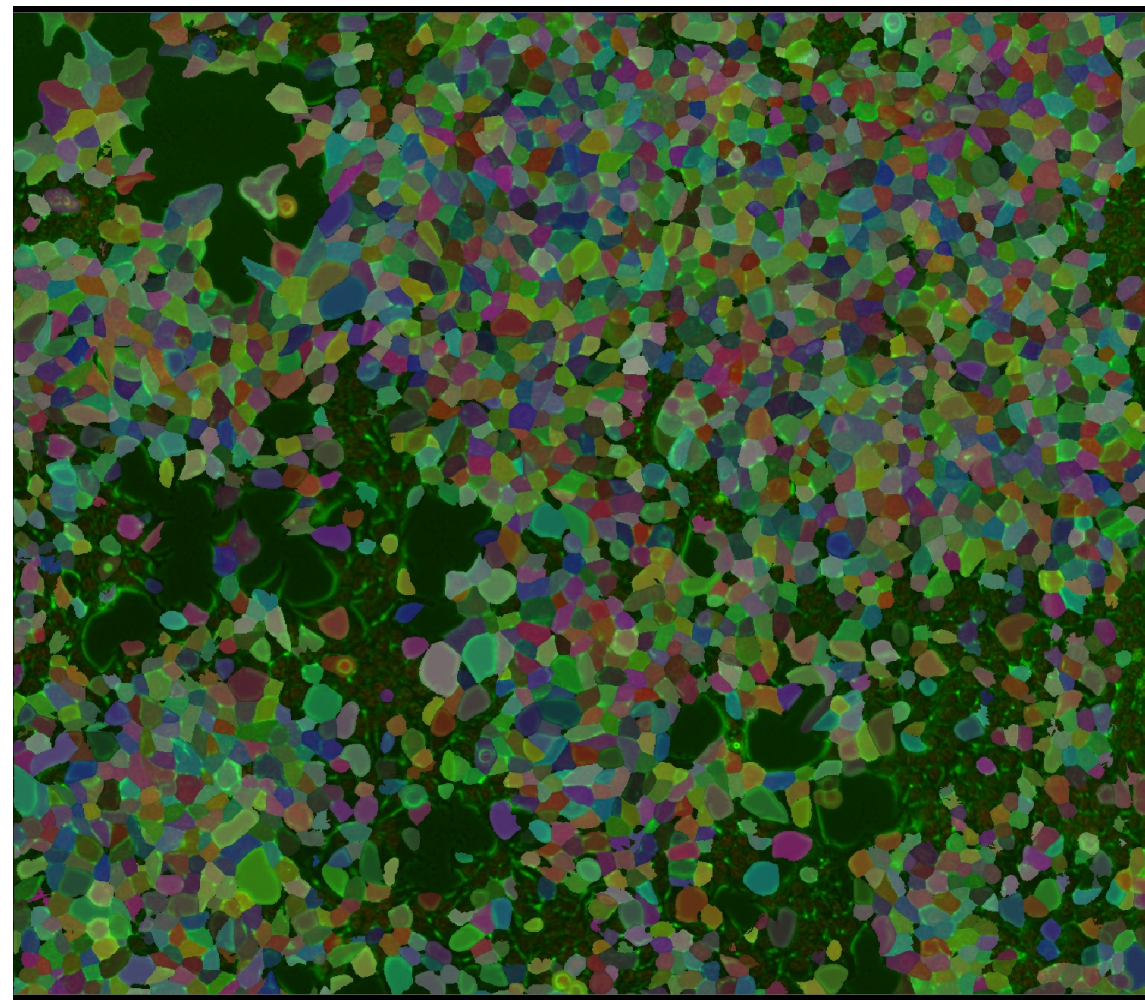

Supplement: S2 Fig — (PDF) [file pcbi.1012271.s002.pdf]

A

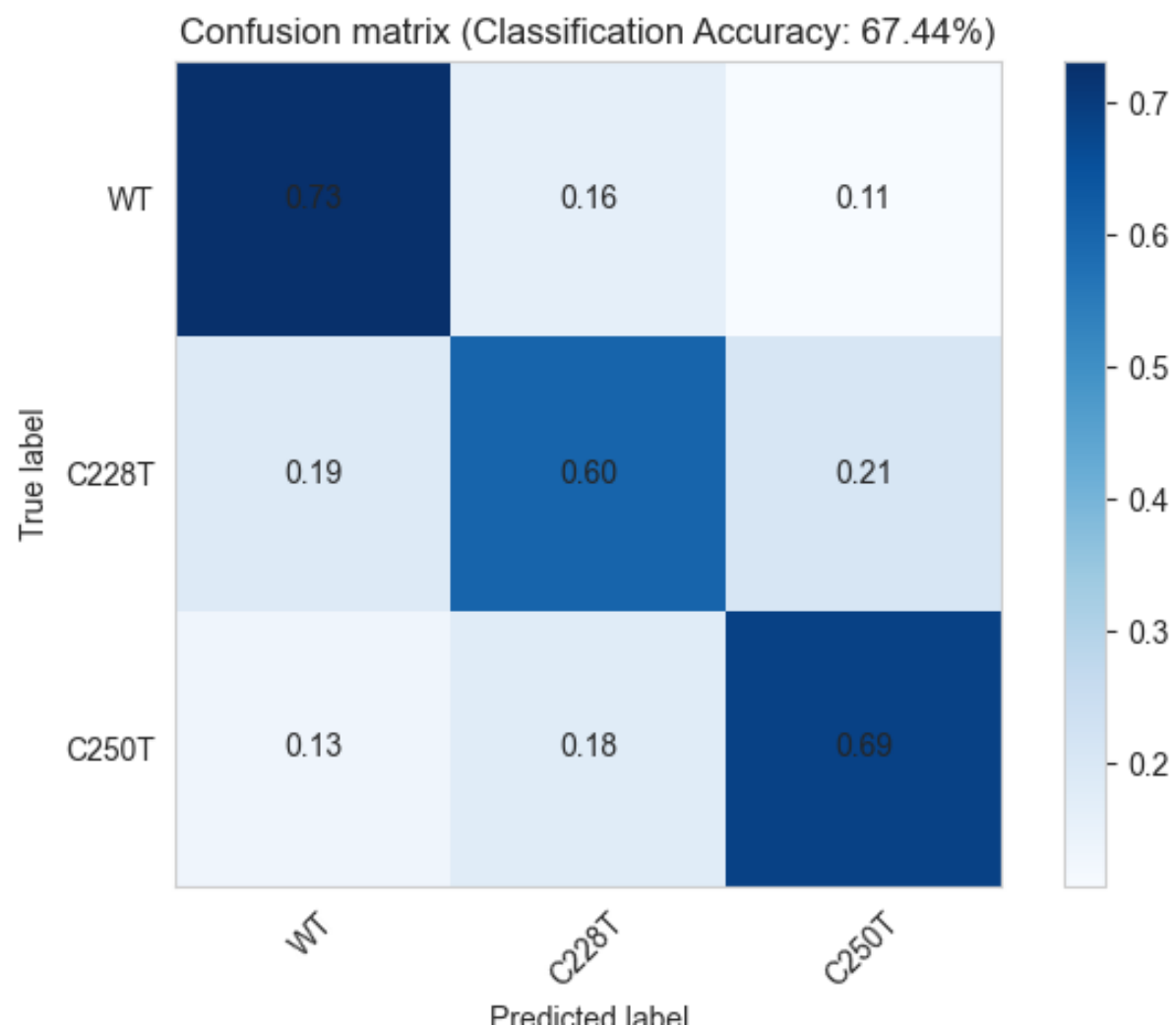

B

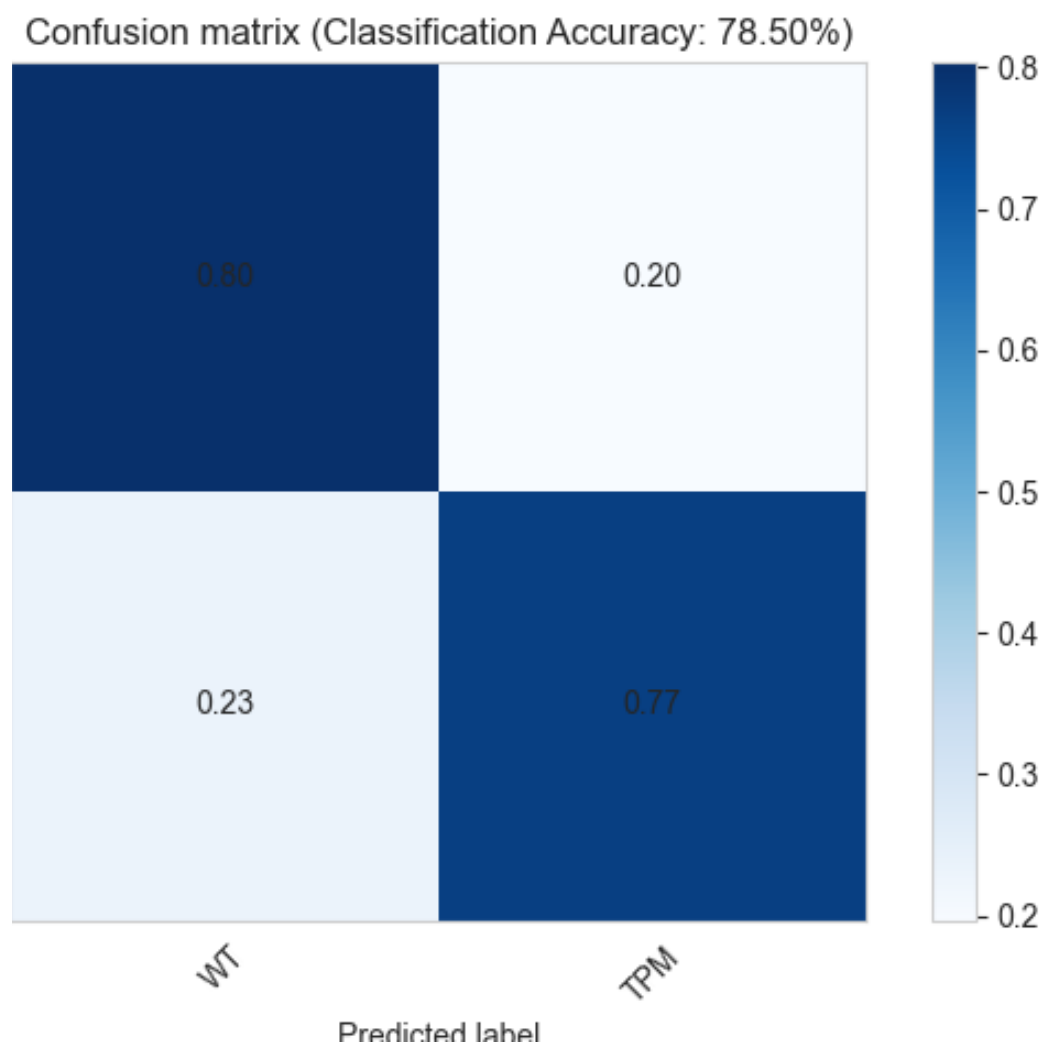

Supplement: S3 Fig — (PDF) [file pcbi.1012271.s003.pdf]

A

TPM

Classification report

0.97

0.93

0.95

WT

0.93

0.97

0.95

Precision

Recall  
Metrics

F1-score

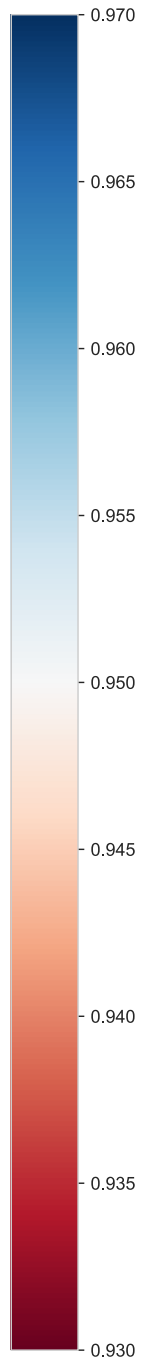

B

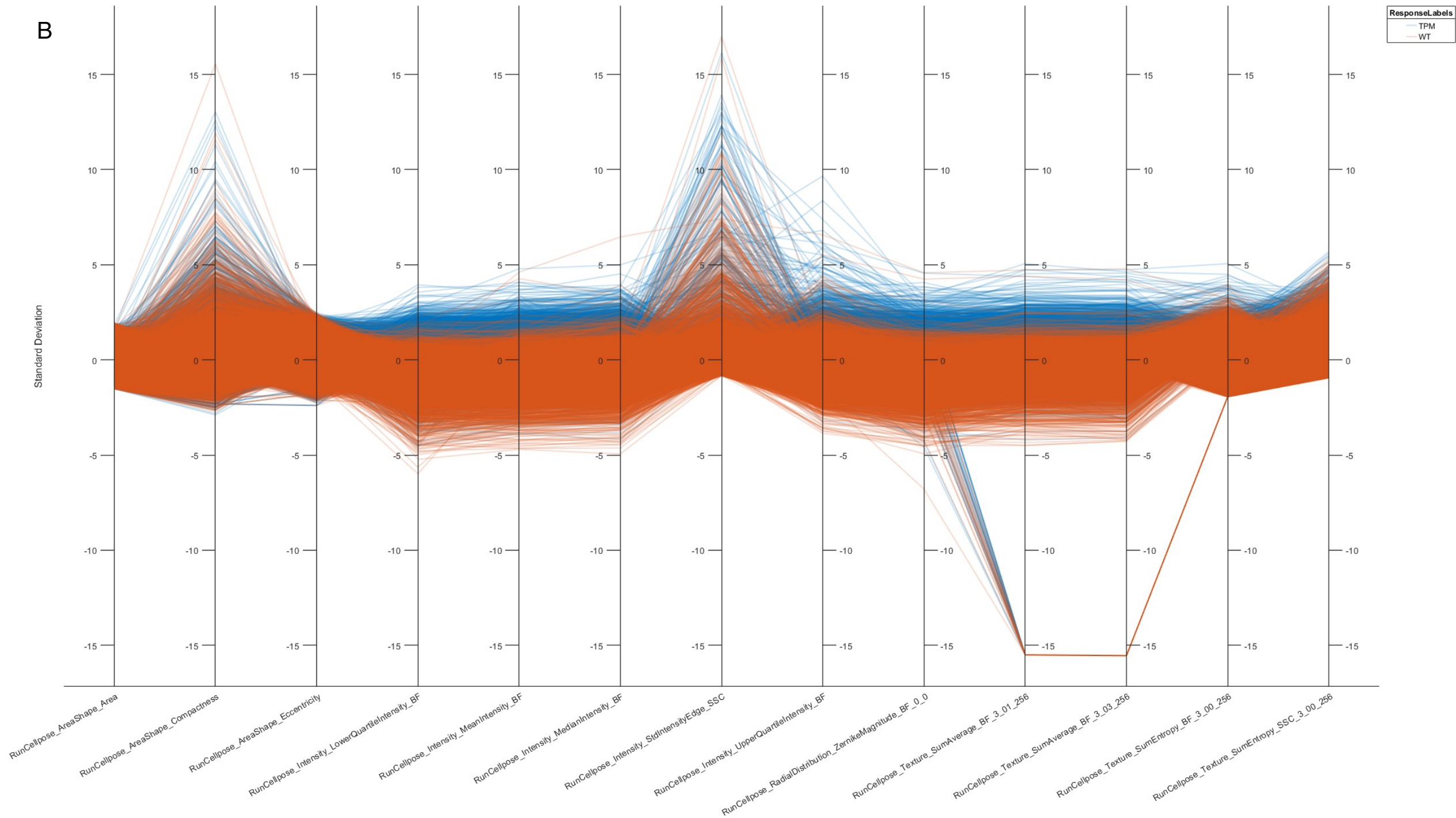

Supplement: S5 Fig — (PDF) [file pcbi.1012271.s005.pdf]

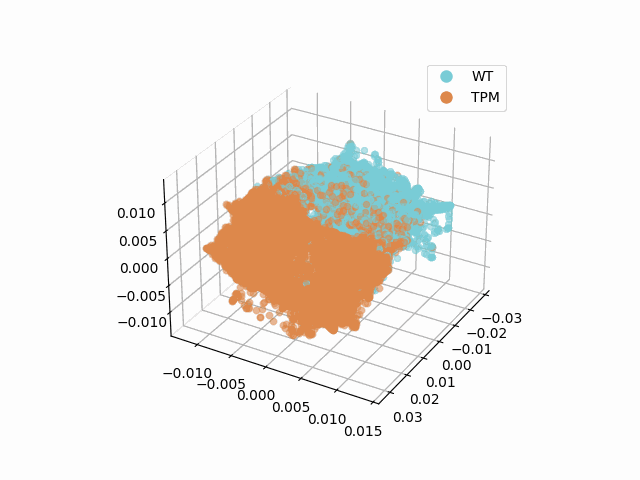

Supplement: S4 Movie — (GIF) [file pcbi.1012271.s010.gif]

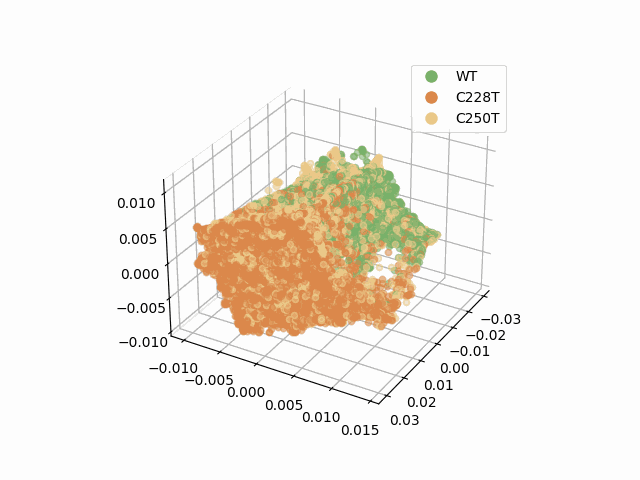

Supplement: S6 Movie — (GIF) [file pcbi.1012271.s012.gif]
