# Supplementary material for: Using deep learning to decipher the impact of telomerase promoter mutations on the dynamic metastatic morpholome
Source: PLoS Comput Biol. 2024 Jul 30;20(7):e1012271. doi: 10.1371/journal.pcbi.1012271 (PMC11288469; doi:10.1371/journal.pcbi.1012271)
Supplement: S4 Fig — (PDF) [file pcbi.1012271.s004.pdf]

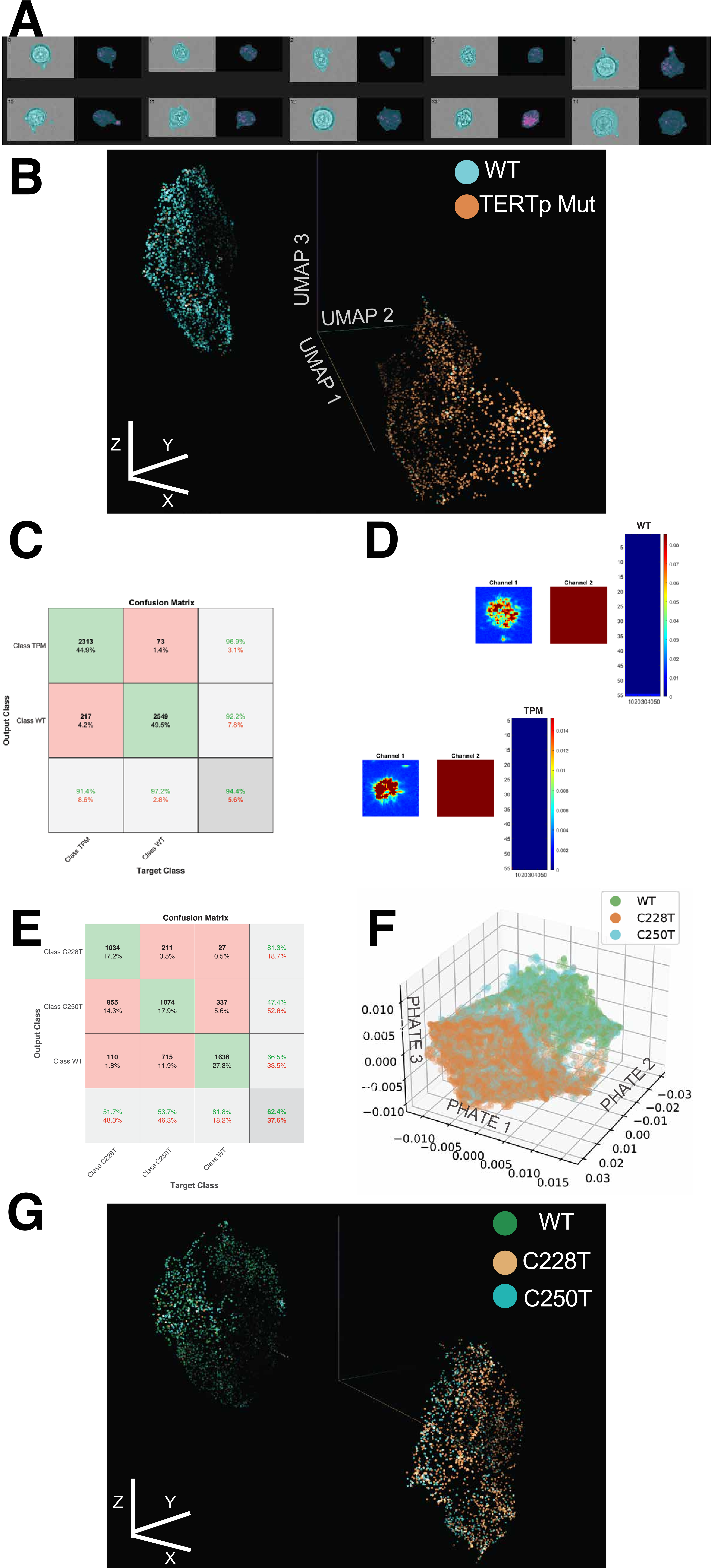
A
B
WT
TERTp Mut
UMAP 3
UMAP 2
UMAP 1
Z
Y
X
C
Confusion Matrix
Class TPM
Class WT
Output Class
2313
44.9%
73
1.4%
96.9%
3.1%
217
4.2%
2549
49.5%
92.2%
7.8%
91.4%
8.6%
97.2%
2.8%
94.4%
5.6%
Class TPM
Class WT
Target Class
D
WT
Channel 1
Channel 2
TPM
Channel 1
Channel 2
E
Confusion Matrix
Class C228T
Class C250T
Class WT
Output Class
1034
17.2%
211
3.5%
27
0.5%
81.3%
18.7%
855
14.3%
1074
17.9%
337
5.6%
47.4%
52.6%
110
1.8%
715
11.9%
1636
27.3%
66.5%
33.5%
51.7%
48.3%
53.7%
46.3%
81.8%
18.2%
62.4%
37.6%
Class C228T
Class C250T
Class WT
Target Class
F
WT
C228T
C250T
PHATE 1
PHATE 2
PHATE 3
G
WT
C228T
C250T
Z
Y
X
